# Biological conversion of methane to putrescine using genome-scale model-guided metabolic engineering of a methanotrophic bacterium *Methylomicrobium alcaliphilum* 20Z

**DOI:** 10.1186/s13068-019-1490-z

**Published:** 2019-06-15

**Authors:** Linh Thanh Nguyen, Eun Yeol Lee

**Affiliations:** Department of Chemical Engineering, Kyung Hee University, Yongin-si, Gyeonggi-do 17104 Republic of Korea

**Keywords:** Methane, Methanotroph, *Methylomicrobium alcaliphilum* 20Z, Putrescine, Metabolic engineering

## Abstract

**Background:**

Methane is the primary component of natural gas and biogas. The huge abundance of methane makes it a promising alternative carbon source for industrial biotechnology. Herein, we report diamine compound, putrescine, production from methane by an industrially promising methanotroph *Methylomicrobium alcaliphilum* 20Z.

**Results:**

We conducted adaptive evolution to improve putrescine tolerance of *M. alcaliphilum* 20Z because putrescine highly inhibits the cell growth. The evolved strain 20ZE was able to grow in the presence of 400 mM of putrescine dihydrochloride. The expression of linear pathway ornithine decarboxylase genes from *Escherichia coli* and *Methylosinus trichosporium* OB3b allowed the engineered strain to produce putrescine. A higher putrescine titer of 12.44 mg/L was obtained in the strain 20ZE-pACO with ornithine decarboxylase from *M. trichosporium* OB3b. For elimination of the putrescine utilization pathway, spermidine synthase (MEALZ_3408) was knocked out, resulting in no spermidine formation in the strain 20ZES1-pACO with a putrescine titer of 18.43 mg/L. Next, a genome-scale metabolic model was applied to identify gene knockout strategies. Acetate kinase (MEALZ_2853) and subsequently lactate dehydrogenase (MEALZ_0534) were selected as knockout targets, and the deletion of these genes resulted in an improvement of the putrescine titer to 26.69 mg/L. Furthermore, the putrescine titer was improved to 39.04 mg/L by overexpression of key genes in the ornithine biosynthesis pathway under control of the pTac promoter. Finally, suitable nitrogen sources for growth of *M. alcaliphilum* 20Z and putrescine production were optimized with the supplement of 2 mM ammonium chloride to nitrate mineral salt medium, and this led to the production of 98.08 mg/L putrescine, almost eightfold higher than that from the initial strain. Transcriptome analysis of the engineered strains showed upregulation of most genes involved in methane assimilation, citric acid cycle, and ammonia assimilation in ammonia nitrate mineral salt medium, compared to nitrate mineral salt medium.

**Conclusions:**

The engineered *M. alcaliphilum* 20ZE4-pACO strain was able to produce putrescine up to 98.08 mg/L, almost eightfold higher than the initial strain. This study represents the bioconversion of methane to putrescine—a high value-added diamine compound.

**Electronic supplementary material:**

The online version of this article (10.1186/s13068-019-1490-z) contains supplementary material, which is available to authorized users.

## Background

Putrescine (1,4-diaminobutane) is a four-carbon diamine found in a wide range of organisms because it is necessary for cell growth and proliferation [1]. Putrescine has many applications in pharmaceuticals, agrochemicals, and surfactants. In chemical industry, putrescine is a chemical platform monomer used for the synthesis of high-performance bioplastic nylon-4,6 that combines the benefits of a high melting point and excellent chemical resistance [2]. There is a high demand for putrescine (about 10,000 tons per year in Europe), and this demand is predicted to increase [3]; thus, putrescine production has received significant attention. There are significant environmental and economic issues associated with the chemical synthesis routes, which requires petrochemical-based raw materials, harsh conditions, and expensive catalyst systems [4, 5]. Therefore, the development of a biotechnological process for the production of putrescine has become attractive.

To date, putrescine has been successfully produced from sugar by metabolic engineering of *Escherichia coli* and *Corynebacterium glutamicum*. Qian et al. reported a metabolically engineered *E. coli* K12 W3110 that produces putrescine in a glucose minimal medium [6]. The ornithine pool was enhanced by overexpression of the ornithine biosynthesis pathway and deletion of the putrescine degradation and utilization pathway. In addition, the activity of a compete pathway conversion of ornithine to arginine was reduced and ornithine decarboxylase was overexpressed. The final strain was able to accumulate 1.68 g/L of putrescine with a yield of 0.166 g/g glucose in a shake flask culture and 24.2 g/L with a productivity of 0.75 g/L h in fed-batch fermentation [6]. Metabolic engineering of *C. glutamicum* for putrescine production has also been reported with a yield from glucose of 0.26 g/g in a flask culture [7] and 0.166 g/g in a fed-batch fermentation [8, 9].

Methane and methanol are one-carbon (C1) substrates that have shown great potential as alternative substrates for biomanufacturing of chemicals and fuels [4, 10, 11]. Utilization of C1 substrates can reduce greenhouse gases and circumvent the social issue of using sugar for making chemicals and fuels. Methane is the cheapest carbon source based on the price per carbon. Thus, there is an increasing demand to convert methane to high value-added products using engineered strains. In recent years, methanotrophic bacteria have become favorable platform strains for industrial biotechnology. A methane- and methanol-utilizing strain, *Methylomicrobium alcaliphilum* 20Z, which can utilize C1 substrates as a sole carbon and energy source [12], has become an attractive model strain due to its advantages: whole genome sequenced, active in various physicochemical conditions (pH, temperature, salinity), and existing genetic tools for genetic manipulation (gene transfer, gene knockout, and replicable vector) [13].

Recently, two genome-based metabolic models of methane oxidation strains have been published [14, 15]. The genome-scale metabolic models with reconstruction of C1-carbon utilization pathway in *M. buryatense* 5GB1 and *M. alcaliphilum* 20Z provide a useful tool to obtain engineering strategies for desired products. Computational strain design has successfully improved 2,3-butandiol production using the genome-scale metabolic model of *M. alcaliphilum* 20Z [16]. A systematic approach for putrescine production has also been reported for *C. glutamicum* [17]. However, there is no report on metabolic engineering of methanotrophs using a genome-based metabolic model for methane bioconversion for putrescine production where the effect of nitrogen needs to be considered.

**Fig. 1:**
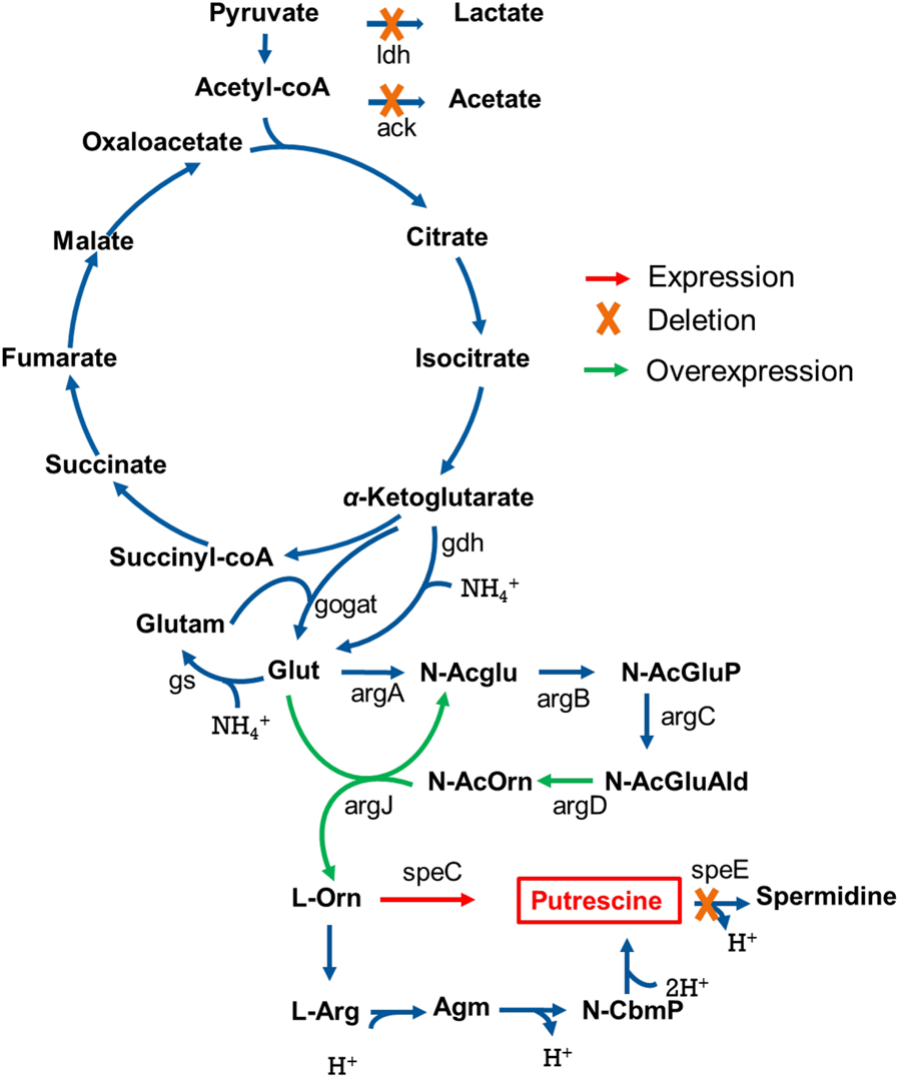
Metabolic engineering of *M. alcaliphilum* 20Z for putrescine production. Red arrows indicate expression reactions. Green arrows indicate overexpression reactions. Yellow X indicates gene deletion. ack, acetate kinase; argA, *N*-acetylglutamate synthase; argB, *N*-acetylglutamate kinase; argC, *N*-acetylglutamylphosphate reductase; argD, acetylornithine aminotransferase; argJ, ornithine acetyltransferase; ldh, lactate dehydrogenase; potE, putrescine transporter; speC, constitutive ornithine decarboxylase; speE, spermidine synthase; speF, inducible ornithine decarboxylase; Agm, agmatine; Glut, glutamate; l-Arg, l-arginine; l-Orn, l-ornithine; *N*-Acglu, *N*-acetylglutamate; *N*-AcGluAld, *N*-acetylglutamate semialdehyde; *N*-AcGluP, *N*-acetylglutamylphosphate; *N*-AcOrn, *N*-acetylornithine; *N*-CbmP, *N*-carbamoylputrescine

In this study, we report a development of engineered *M. alcaliphilum* 20Z strains for the production of putrescine from methane. The engineered *M. alcaliphilum* 20Z strain with inactive putrescine utilization pathway and expression of ornithine decarboxylase converted methane to putrescine. Furthermore, engineering strategies were generated by using the genome-scale metabolic model, which allowed further improvement of putrescine production from methane (Fig. 1).

## Results

### Analysis of putrescine toxicity on *M. alcaliphilum* 20Z

**Fig. 2:**
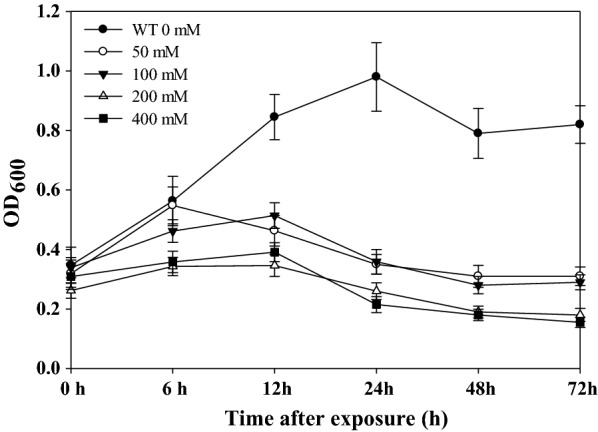
Putrescine tolerance of *M. alcaliphilum* 20Z. Cells were grown at 30 °C in an NMS medium and exposed to various concentration of putrescine dihydrochloride in the exponential growth phase (OD_600_ of 0.4)

In general, putrescine is toxic to microorganisms [18]. Therefore, the impacts of extracellular putrescine inhibition on *M. alcaliphilum* 20Z growth was first examined. *M. alcaliphilum* 20Z was cultured to an exponential phase and exposed to different putrescine concentrations of 50 mM, 100 mM, 200 mM, and 400 mM (Fig. 2). *M. alcaliphilum* 20Z showed significantly reduced growth rates in the presence of 50 mM and higher putrescine concentrations. Approximately, 50 mM of putrescine dihydrochloride (equivalent to 4.4 g/L putrescine) caused cell lysis. Based on this observation, we concluded that low putrescine tolerance could be a main barrier for metabolic engineering of *M. alcaliphilum* 20Z for putrescine production.

### Enhancement of putrescine tolerance of *M. alcaliphilum* 20Z by adaptive laboratory evolution

**Fig. 3:**
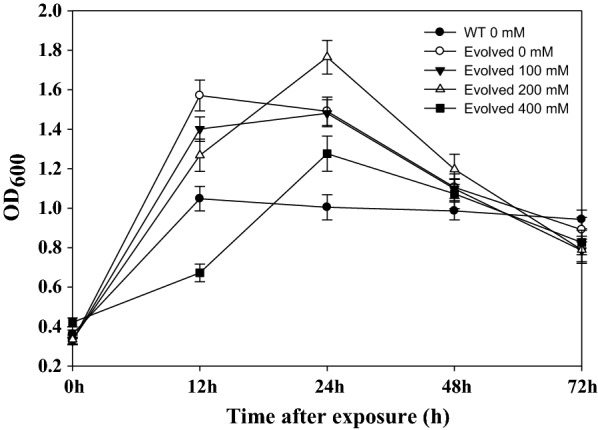
Improvement of putrescine tolerance of *M. alcaliphilum* 20Z by adaptive evolution. Filled circles show the wild-type strain *M. alcaliphilum* 20Z grown in NMS. The open circles show the evolved strain in NMS, while the filled triangles show the evolved strain exposed to 100 mM of putrescine dihydrochloride. The open triangles show the evolved strain exposed to 200 mM of putrescine dihydrochloride, and the filled squares show the evolved strain with 400 mM of putrescine dihydrochloride

Adaptive evolution to improve putrescine tolerance of *M. alcaliphilum* 20Z was first conducted to overcome low putrescine tolerance of *M. alcaliphilum* 20Z. Cells exposed to 100 mM of putrescine dihydrochloride in the tolerance test were incubated in NMS with 0.2% methanol at 30 °C, 230 rpm for 15 days. Then, 2 mL of culture broth were transferred to fresh NMS medium with 0.2% methanol to recover. A long lag phase of 3 days was observed, and active cells were transferred to fresh NMS medium. Cells were grown to an OD_600_ of 0.4 and exposed to 100 mM of putrescine dihydrochloride for 7 days. After 5 replication cycles (approximately more than 65 days in total), a strain with a high tolerance to putrescine was obtained (referred to as the 20ZE strain). The evolved putrescine-tolerant 20ZE strain was able to grow in the presence of 400 mM putrescine dihydrochloride. The growth rate of the evolved strain was comparable to that of wild-type strain (0.089 h^−1^), and the evolved strain showed even slightly better growth rate in the presence of 100 and 200 mM putrescine (0.121 h^−1^ and 0.111 h^−1^, respectively) (Fig. 3). Extension of putrescine tolerance on *M. alcaliphilum* 20Z allowed the evolved strain to be employed for further metabolic engineering for putrescine production from methane.

### Construction of a platform host strain for putrescine production from methane and methanol

Putrescine can be synthesized in vivo via ornithine by constitutive ornithine decarboxylase (speC) or inducible ornithine decarboxylase (speF) and arginine by arginine decarboxylase and agmatinase. *M. alcaliphilum* 20Z possesses alternative agmatine deiminase, which hydrolyzes agmatine to ammonia and *N*-carbamoylputrescine. The latter is subsequently hydrolyzed to putrescine, ammonia, and carbon dioxide by *N*-carbamoylputrescine amidohydrolase [9]. In addition, *M. alcaliphilum* 20Z also possesses a putrescine utilization pathway, which converts putrescine to spermidine by spermidine synthase (speE). There are five genes predicted as spermidine synthase, of which three genes have lengths of about 516 (MEALZ_1869), 825 (MEALZ_1164), and 856 (MEALZ_3010) amino acids, respectively. The two other genes are very similar to spermidine synthases of *E. coli*, which have protein lengths of 282 (speE1-MEALZ_3408) and 267 (speE2-MEALZ_3304) amino acids, respectively. Therefore, we tried to inactivate these two genes with similar protein lengths of *E. coli* spermidine synthase, and test whether putrescine could be accumulated in the mutant *M. alcaliphilum* 20Z strain. The spermidine synthase gene (speE1) was knocked out through unmarked allelic exchange using a sucrose counter-selection system [19]. Vector pCM433 was constructed with two flanking homology regions upstream and downstream of MEALZ_3408, resulting in vector pCSE1. Vector pCSE1 was introduced into strain 20ZE by electroporation to be integrated into the genome of 20ZE by single crossover. The single-crossover recombinants were transferred to fresh NMS medium containing 2.5% (w/v) of sucrose and were selected for the mutants that excised the vector by PCR using the primer outside the flanking regions. The speE1 mutant was obtained as strain 20ZES1, which accumulated 0.35 mg/L of putrescine after 96 h cultivation. The spermidine concentration in the culture broth was also analyzed, and there was no spermidine accumulation. This means that the knockout of the spermidine synthase (MEALZ_3408) blocked the conversion of putrescine to spermidine. Spermidine and other polyamines are involved in critical physiological processes in bacteria such as cell growth, biofilm formation, stress response, and proliferation [20], but, interestingly, spermidine was proved to be not essential for the growth of *E. coli* and *Saccharomyces cerevisiae* [21, 22]. Obviously, inactivation of putrescine degradation and utilization pathway in *E. coli* resulted in a higher putrescine titer [6]. Similarly, inactivation of the putrescine utilization pathway allowed putrescine to be accumulated in the engineered strain 20ZES1.

To increase putrescine production in the *M. alcaliphilum* 20Z, ornithine decarboxylase was expressed to directly convert ornithine to putrescine. Constitutive ornithine decarboxylase (speCEc) and a native putrescine transporter (potE) from *E. coli* K12 were amplified and cloned into an IncP-based broad host-range vector pAWP89 under control of a pTac promoter, resulting in a pACE vector. An ornithine decarboxylase (speCOb) from *M. trichosporium* OB3b, a type II methanotroph, was also cloned into the pAWP89 vector, resulting in vector pACO. Moreover, the codon adaptation index (CAI) of those genes (for predicting gene expression level) was calculated based on a *M. alcaliphilum* 20Z codon usage table. The CAI values of speCEc and speCOb were relatively high at 0.73 and 0.78, respectively. Vectors pACE and pACO were successfully transformed into wild-type 20Z by electroporation, resulting in strains 20Z-pACE and 20Z-pACO, respectively. Recombinant strains were cultured in a shake flask containing 50 mL of NMS medium with 50% (v/v) methane. Putrescine accumulation in the supernatant was analyzed by HPLC as described above. After 144 h of incubation, recombinant strain 20Z- pACE produced 2.27 ± 0.42 mg/L of putrescine, while the 20Z-pACO strain produced 12.44 ± 0.86 mg/L of putrescine, approximately five times higher. Enzyme ornithine decarboxylase (speCOb) from *M. trichosporium* OB3b showed high activity even with an alkali pH in the culture medium. Vector pACO would be used for further putrescine production experiments.

**Fig. 4:**
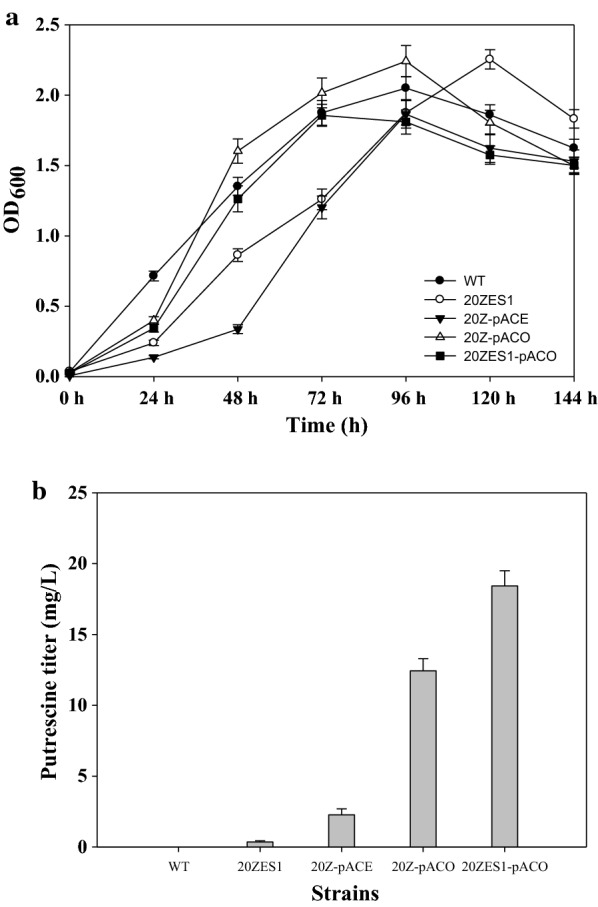
**a** Growth of wild-type and engineered strain *M. alcaliphilum* 20Z. **b** Putrescine accumulation of engineered strain *M. alcaliphilum* 20Z with spermidine synthase knockout and strain harboring pACE and pACO vector after 144 h of cultivation. The data are represented as the mean ± SD (*n* = 3)

The methylated vector pACO harvested from 20Z strains was easily transformed to strain 20ZES1 with high efficiency, resulting in strain 20ZES1-pACO. Interestingly, strain 20ZES1 produced 18.43 ± 1.08 mg/L putrescine in shake flasks after 144 h (Fig. 4). This production increased by 32.5% compared to the wild-type strain harboring vector pACO.

### Metabolic engineering strategy for enhancing putrescine production based on *in silico* simulation

Genome-scale metabolic model can be employed to identify gene knockout strategies to optimize the metabolic pathway for the target product. Genome-scale metabolic models have successfully been applied to *M. alcaliphilum* 20Z [14, 16]. OptGene is computationally efficient, and it has been successfully applied (i) in *Saccharomyces cerevisiae* to improve succinate production by 30-fold [23], (ii) in *Synechocystis* to allow for growth-coupled biofuel production [24], and (iii) in *M. alcaliphilum* 20Z for improving the production of 2,3-butandiol [16]. OptGene was performed to identify gene deletion strategies to improve the biomass production-coupled yield (BPCY) as well as the yield of putrescine. In the OptGene algorithm, reaction knockouts were randomly introduced to obtain a mutant population [25]. The objective function was calculated using minimization of metabolic adjustment (MOMA), which identified the closest flux point to the wild-type point making it compatible with the gene deletion constraint [26].

Maximization of the specific growth rate and secretion of putrescine was used as the objective function with flux balance analysis (FBA) to predict the phenotype of the wild-type strain. The maximum theoretical yield and maximum productivity of putrescine production from methane were computed to be 0.579 g/g methane and 0.937 mmol/gDCW/h. Putrescine production is not favorable at the optimal biomass growth rate, as indicated by the FBA using maximization of the specific growth rate as the objective function. This resulted in no putrescine accumulation.

An *in silico* evolutionary programming-based method was performed using OptGene to optimize putrescine production. OptGene-derived mutations that increased BPCY for putrescine production were generated and are listed in Additional file 1: Table S1. The most common knockouts for putrescine production were acetate kinase (ACKr), serine hydroxymethyltransferase (glyA), and methylenetetrahydrofolate dehydrogenase (MTHFD). GlyA and MTHFD are two genes that evolved in the tetrahydromethanopterin (H_4_MPT) pathway and serine cycle. Through this pathway, formaldehyde was oxidized to formate, which played a role in formaldehyde detoxification and provided NADH for methane oxidation [27]. Knockout of these two genes was not favorable for strain growth in methane. Moreover, the predicted flux value via the H_4_MPT pathway was small, i.e., 0.1 compared with 11.6 via the RuMP pathway in *M. alcaliphilum* 20Z [14]. Knockout of ACKr gave a BPCY of 0.0027 with the highest productivity of 0.66 mmol/gDCW/h of putrescine and a yield of 0.408 g-(putrescine)/g-CH_4_, which is approximately 70% of the theoretical yield. The maximum specific growth rate was predicted as 0.032 h^−1^. Thus, it was selected as a knockout target. Subsequently, lactate dehydrogenase (LDH) was also selected as a promising target to enhance putrescine production. With the knockout mutant of ACKr, the flux redistribution toward putrescine formation from acetyl-CoA can be improved, because of the decrease in the flux from acetyl-CoA to acetate.

**Fig. 5:**
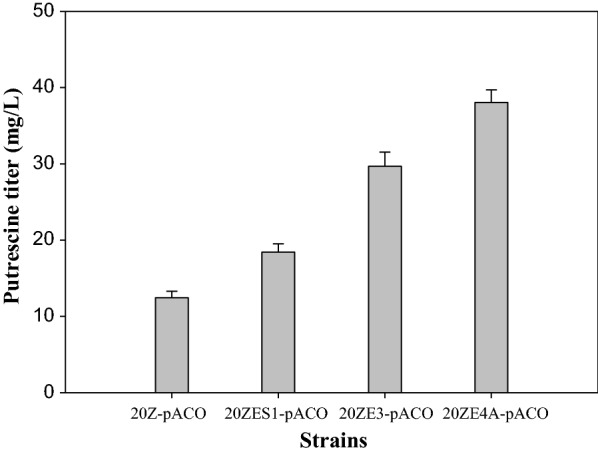
Putrescine improvement in engineered strains. The data are represented as the mean ± SD (*n* = 3)

A triple mutant strain was successfully generated using sucrose counter-selection, resulting in strain 20ZE3. Putrescine production by the mutant strain was examined through the transformation of vector pACO to construct strain 20ZE3-pACO. Strain 20ZE3-pACO was able to accumulate 26.69 ± 1.86 mg/L of putrescine after 144 h of cultivation, which is a 37.03% improvement compared to the 20ZES1-pACO strain (Fig. 5).

Furthermore, improving the ornithine pool (i.e., the direct precursor of putrescine) would be necessary to improve the putrescine titer. Ornithine was synthesized from glutamate by five sequential catalytic reactions catalyzed by *N*-acetylglutamate synthase (encoded by argA), acetylglutamate kinase (argB), *N*-acetylglutamate semialdehyde dehydrogenase (argC), and *N*-acetylornithine transaminase (argD) to yield *N*-acetylornithine. Ornithine was formed in a recycling pathway by bifunctional ornithine acetyltransferase (argJ), which catalyzed *N*-acetylglutamate from glutamate, and ornithine by transacetylation between *N*-acetylornithine and glutamate. *M. alcaliphilum* 20Z possesses the recycling pathway, which was recognized as an efficiency pathway for production of an ornithine-derived product such as putrescine; the recycling pathway was an efficient pathway compared to the linear pathway catalyzed by acetylornithine deacetylase (argE) found in *E. coli*, type II methanotrophs, and other species, which converted acetylornithine to ornithine and generated acetate as an intermediate product [28]. These genes are located in five different loci. Plasmid-based overexpression of argCJBDFRGH or argJ alone in *C. crenatum* SYPA 5-5 also led to enhanced arginine production, the downstream product of ornithine [29]. Overexpression of argJ increases flux from glutamate toward an ornithine biosynthesis pathway and efficient conversion of acetylornithine to ornithine.

Protein–protein association networks with speC were also investigated [30]. The interaction of argD with speC was found in a characterized methylotrophic strain, *Methylobacterium extorquens* AM1 [31]. Thus, argD and argJ, two native genes in *M. alcaliphilum* 20Z, were selected as targets for overexpression to improve the ornithine pool. *N*-acetylornithine transaminase (argD) and ornithine acetyltransferase (argJ) were cloned into pAWP89 under the control of a pTac promoter. Two flanking regions in the genome of *M. alcaliphilum* 20Z were constructed into vector pCM351 along with the pTac-argDJ fragment. Flank F2 was inserted into the SacI site, resulting in vector pCM351-F2. Flank F1 and pTac-argDJ fragments were inserted in the *Eco*RI and *Not*I sites to generate vector pCAR2. Vector pCAR2 was introduced into 20ZE3, and a double crossover allelic exchanged mutant 20ZE4A with the genotype ΔldhΔackΔspeE1::argDJ was successfully obtained. In these strains, putrescine production was improved by 21.08% and 51.57% compared to 20ZE3-pACO and 20ZES1-pACO, respectively. A maximized titer of 39.04 ± 1.35 mg/L (approximately three times higher than 20Z-pACO) was obtained (Fig. 5).

### Enhanced production of putrescine with nitrogen source optimization

Although ammonia was considered to be the best inorganic nitrogen source for *E. coli* because it provided the fastest growth rate [32], it is not widely used for culturing methanotrophs. Currently, nitrate is the main nitrogen source for culturing methanotrophs. Nitrate is subsequently reduced to nitrite and finally to ammonia by nitrate reductase and nitrite reductase [33]. Ammonia was then assimilated by glutamate dehydrogenase (GDH) or the glutamine synthetase/glutamate synthase (GS/GOGAT) pathway. Nitrate assimilation is an energetically expensive process, which is not favorable for growth of bacteria and for glutamate-derived products. In addition, the GDH pathway was stimulated in the presence of ammonia, leading to direct assimilation of ammonia to glutamate. Therefore, we investigated the effect of different nitrogen sources on the growth of *M. alcaliphilum* 20Z and putrescine production from methane.

Wild-type and the engineered strains 20ZE4A-pACO were grown in nitrate mineral salt medium (NMS) with 10 mM of potassium nitrate and in an ammonium mineral salt medium (AMS) with various concentrations of ammonium chloride: 1 mM, 2 mM, 5 mM, 10 mM, and 20 mM. Unfortunately, no growth of *M. alcaliphilum* 20Z was observed in AMS media at any concentration of ammonium chloride. Due to the lack of substrate specificity, methane monooxygenases oxidized ammonia to hydroxylamine, which led to an incompatibility between methane oxidation and ammonia oxidation. In addition, ammonia has potential toxicity to microorganism under high pH condition [34]. Cultivation of wild-type and engineered strains in lower pH was also conducted. The wild-type and engineered strains were slowly grown in NMS at neutral pH, but they did not grow in AMS at any pH (data not shown). Therefore, ammonia toxicity is mainly caused by the incompatibility of ammonia oxidation and methane oxidation and toxicity of hydroxylamine—an ammonia oxidation product [35, 36]. Ammonia is not a suitable nitrogen source for growth in methane. However, an appropriate amount of ammonium chloride supplement during growth in NMS can enhance putrescine production. Growth of *M. alcaliphilum* 20Z in ammonia nitrate mineral salt medium (ANMS) with various concentrations of ammonium chloride was also examined.

**Fig. 6:**
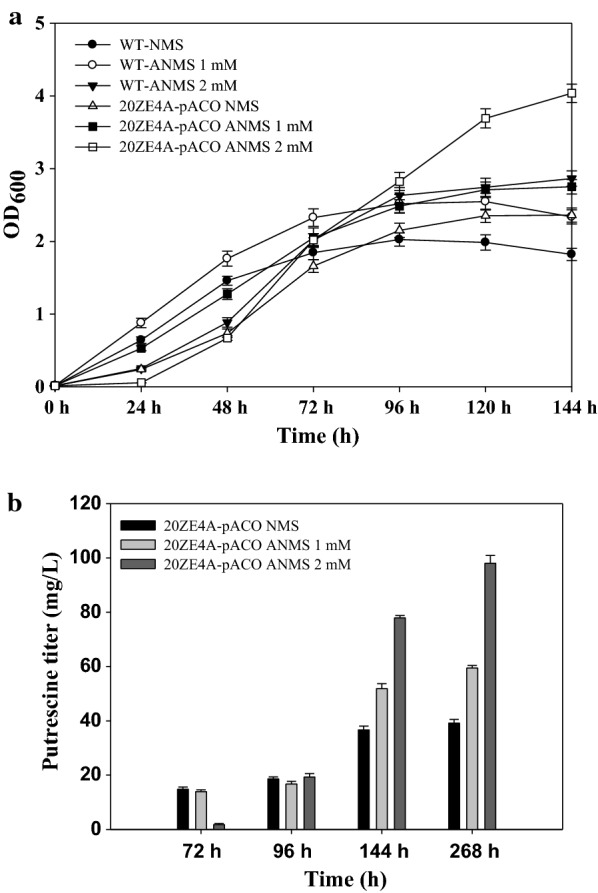
**a** Growth of *M. alcaliphilum* 20Z and engineered strains in NMS and ANMS media. **b** Putrescine production by the engineered strains 20ZE4A-pACO in NMS and ANMS with different concentrations of ammonium chloride over time

The performance of wild-type and engineered strains 20ZE4A-pACO in ANMS with various concentrations of ammonium chloride (1 mM, 2 mM, 5 mM of NH_4_Cl) was examined. With the supplement of 1 mM ammonium chloride, the wild-type and the engineered strains 20ZE4A-pACO showed higher growth rate in comparison with growth in NMS. With the supplement of 2 mM ammonium chloride, the growth of wild-type and engineered strains showed longer lag phase in the first 48 h of growth. However, after 96 h, the engineered strains reached 1.12-fold higher optical density than wild type, and the engineered strain reached highest optical density (OD_600_ = 4.0) compared to that of wild type (OD_600_ = 2.5) in flask culture (Fig. 6a). However, no growth of either wild-type or the engineered strain was observed with 5 mM of ammonium chloride.

After 268 h of cultivation, the engineered strain 20ZE4A-pACO produced 39.14 ± 1.35 mg/L of putrescine in an NMS medium. On the other hand, this engineered strain was able to produce 59.46 ± 0.92 and 98.08 ± 2.86 mg/L of putrescine in ANMS with 1 mM and 2 mM of ammonium chloride, respectively (Fig. 6b). In ANMS with 2 mM of ammonium chloride, the highest titer of 98.08 mg/L putrescine with a productivity of 2.9 nmol/gDCW/h and a yield of 0.0276 g-(putrescine)/g-CH4 was obtained. This was approximately 2.5 times higher than that obtained in NMS and is the highest titer of putrescine production from methane.

### Analysis of gene expression level in response to change of nitrogen sources

**Fig. 7:**
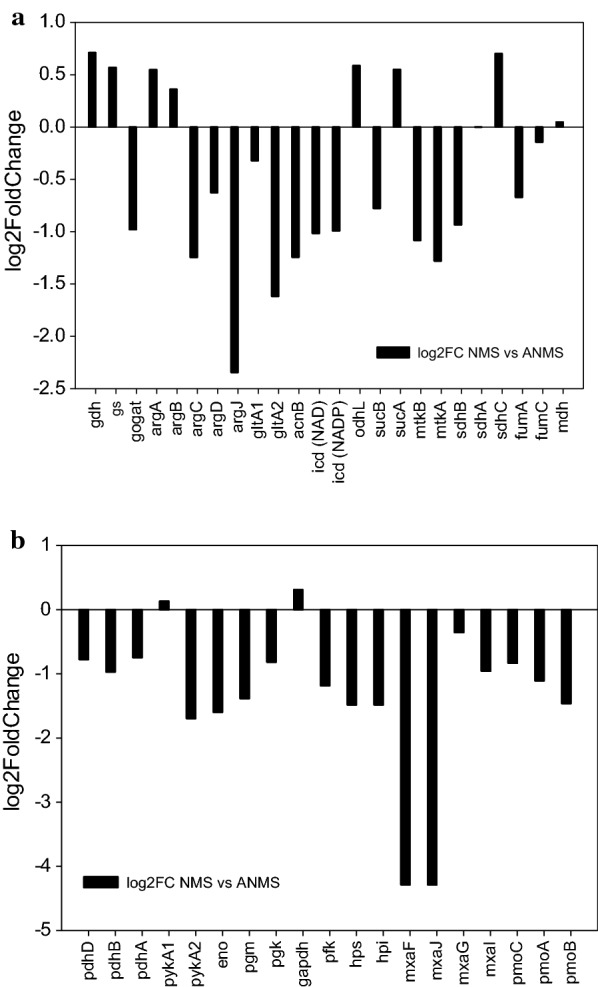
Different gene expression of genes involves in **a** TCA cycle and ammonia assimilation pathway and **b** central metabolic pathway, estimated as log2FoldChange of NMS vs ANMS (black bar)

For further understanding how gene expression is regulated in response to the change of nitrogen sources, transcriptome shotgun sequencing (RNA-seq) of *M. alcaliphilum* 20Z strain 20ZE4A-pACO cultured in ANMS and NMS was analyzed. Different expression levels of genes involved in ammonia assimilation, TCA cycle (Fig. 7a), and central metabolic pathway (Fig. 7b) were estimated as logarithm base twofold change (log2FoldChange) of gene expression level in NMS versus ANMS. Log2FoldChange < 0 of the given gene means upregulation of this gene in ANMS compared to NMS.

Among genes involved in ammonia assimilation in ANMS, only GOGAT was upregulated, while both GDH and GS were downregulated. Genes involved in ornithine biosynthesis pathway, argC, argD, and argJ, were highly upregulated in ANMS, especially, argJ was strongly upregulated with five-fold changes. Genes of the upstream of α-ketoglutarate dehydrogenase in TCA cycle were highly upregulated in ANMS, and only slight changes were observed in the downstream genes with some downregulation. Genes in the EMP pathway and methane assimilation were mostly upregulated in ANMS. Interestingly, methanol dehydrogenase large subunit mxaF and a downstream gene mxaJ were 19-fold upregulated in ANMS compared to NMS. Genes in methane assimilation, upstream reactions of TCA cycle, ammonia assimilation (GOGAT)n and ornithine biosynthesis pathway were significantly upregulated in ANMS. It can be inferred that small amount of ammonia, which reduced the incompatible inhibition of methane oxidation and ammonia oxidation, could be directly assimilated into glutamate. The saved energy consumption due to the decrease of nitrate assimilation might enhance the methane assimilation, together with 19-fold upregulation of methanol dehydrogenase large subunit in ANMS compared to NMS. As a result, a high titer of putrescine along with high growth rate and cell density was obtained in the ANMS medium.

## Discussion

Methanotrophs with the ability of using methane as the sole carbon and energy source are becoming promising strains for methane bioconversion. *M. alcaliphilum* 20Z, a haloalkali-tolerant methanotroph, is an industrially promising biocatalyst for the conversion of methane to value-added products due to a well-characterized central metabolic pathway, rapid growth rates, high cell density cultivation, and genetic tools [37]. In addition, *M. alcaliphilum* 20Z possesses an efficient recycling pathway for the production of ornithine-derived product, compared to the linear ornithine biosynthesis pathway of type II methanotrophs, which forms acetate as the bypass product.

In this study, we employed *M. alcaliphilum* 20Z for the conversion of methane to an important industrial chemical, putrescine. However, compared to engineered *E. coli*, *M. alcaliphilum* 20Z was very sensitive to putrescine. *E. coli* can grow well in the presence of 250 mM putrescine dihydrochloride (22 g/L putrescine), and the growth rate significantly decreased in the presence of 500 mM putrescine dihydrochloride (44 g/L putrescine) [6]. The effect of putrescine on the growth of *C. glutamicum* was also reported. *C. glutamicum* was able to grow in the presence of up to 500 mM putrescine dihydrochloride, but the growth rate was reduced (34%) at 740 mM putrescine dihydrochloride [9]. Therefore, we have conducted the adaptive evolution to enhance putrescine tolerance of *M. alcaliphilum* 20Z. The evolved strain was able to grow in the presence of 400 mM putrescine dihydrochloride.

We constructed an IncP-based broad host-range plasmid for expression of ornithine decarboxylase from a well-known source, *E. coli,* and from a type II methanotroph, *M. trichosporium* OB3b, driven by a tac promoter. The expression of ornithine decarboxylase from *M. trichosporium* OB3b showed higher activities at an alkali pH in a cultivation medium of *M. alcaliphilum* 20Z.

In addition, we inactivated the putrescine utilization pathway to accumulate putrescine in *M. alcaliphilum* 20Z. *M. alcaliphilum* 20Z possesses five genes predicted to be associated with spermidine synthase. However, the knockout of one spermidine gene MEALZ_3408 completely blocked spermidine formation in the mutant strain (data not shown). Gene MEALZ_3304 could be associated with spermine synthase, which converts spermidine to spermine. We also found that a small amount of putrescine accumulation in the culture medium promoted the growth of *M. alcaliphilum* 20Z.

A genome-scale metabolic model of *M. alcaliphilum* 20Z and its application have been recently published [14]. By employing the genome-scale metabolic model, we present genetic engineering strategies to obtain the engineered *M. alcaliphilum* 20Z strains where putrescine production increased or growth was coupled. The ACKr gene was identified as a potential target for increase in putrescine production. With ACKr knockout, strain 20ZE3-pACO was able to produce 26.69 ± 1.86 mg/L of putrescine. For further improvement of putrescine production, we introduced key genes in the ornithine biosynthesis pathway-argDJ by integration. These genes were driven by tac promoter into the genome of *M. alcaliphilum* 20Z, which led to an improvement of 21.08% with a maximized titer of 39.04 ± 1.35 mg/L.

We further examined the effects of different nitrogen sources on the growth and putrescine production of the wild-type and engineered *M. alcaliphilum* 20ZE4A-pACO strain. Generally, *M. alcaliphilum* 20Z has been cultured with nitrate as the sole nitrogen source. Due to the incompatibility of ammonia oxidation and methane oxidation, methane monooxygenases (which lack substrate specificity) oxidized ammonia to hydroxylamine that inhibits methane oxidation [35, 36]. Nitrate assimilation is an energetically expensive process. However, the assimilation of ammonia via glutamate dehydrogenase, which was employed in energy-limited environment, could be favorable for a glutamate-derived product like putrescine. *M. alcaliphilum* 20Z was unable to grow with ammonia as the sole nitrogen source. We found that the growth of *M. alcaliphilum* 20Z was promoted and putrescine production was also significantly improved with up to 2 mM of ammonium chloride supplement. A maximized titer of 98.08 ± 2.86 mg/L putrescine with a productivity of 2.9 nmol/gDCW/h was obtained in a simple flask culture.

Transcriptome analysis of the engineered strains cultured on mineral salt medium with different nitrogen sources showed upregulation of most genes involved in methane assimilation, TCA cycle, and ammonia assimilation in ammonia nitrate mineral salt medium compared to nitrate mineral salt medium. Despite the incompatibility of ammonia with methane, ammonia could be used as a potential nitrogen source for growth of *M. alcaliphilum* 20Z and production of glutamate-related metabolites.

The maximum titer of putrescine obtained in this study was much lower than sugar-based production [6, 9]. The experimental putrescine yield was 0.0276 g/g-CH_4_, much lower than the yield predicted by Optgene (0.408 g/g-CH_4_), but the experimental specific growth rate of the engineered strain (0.048 h^−1^) in NMS was higher than the maximum specific growth rate predicted by Optgene (0.032 h^−1^). The difference in growth rates between simulated data and experimental data was due to the adaptation to putrescine by engineered strain. To enhance the productivity of putrescine from methane, the remaining obstacles of methane bioconversion including methane mass transfer limitation and methanotrophic methane oxidation efficiency [5, 38, 39] should be solved. In addition to the conventional limitations of methane bioconversion (such as low methane mass transfer, lack of genetic tools, and low carbon conversion efficiency), further studies need to be investigated for engineering methane monooxygenase to allow *M. alcaliphilum* 20Z to grow in ammonia, which can reduce the energy consumption by nitrate assimilation.

## Conclusion

The obligate methanotrophic bacterium *M. alcaliphilum* 20Z has become an attractive microbial platform for methane bioconversion to a value-added product due to the development of genetic tools and new insights into methane assimilation and the central metabolic pathway. In this study, we applied a genome-scale metabolic model to identify the metabolic engineering targets, inactivated the putrescine utilization pathway in *M. alcaliphilum* 20Z, overexpressed the ornithine biosynthesis pathway, and optimized the nitrogen source for putrescine production. The engineered *M. alcaliphilum* 20ZE4A-pACO strain produced putrescine up to 98.08 ± 2.86 mg/L in simple flask culture. To the best of our knowledge, this work represents the first biotechnological application for producing putrescine from methane.

## Methods

### Strains and culture conditions

The bacterial strains and plasmids used in this study are listed in Table 1. Wild-type and engineered *M. alcaliphilum* 20Z strains were cultured in nitrate mineral salt medium (NMS), which contains 1.0 g/L MgSO_4_·7H_2_O, 0.02 g/L CaCl_2_·6H_2_O, 1.0 g/L KNO_3_, 15 g/L NaCl, 2 mL/L trace element solution (Na_2_-EDTA 0.5 g/L, FeSO_4_·7H_2_O 1 g/L, F2-EDTA 0.75 g/L, ZnSO_4_·7H_2_O 0.8 g/L, MnCl_2_·4H_2_O 0.005 g/L, H_3_BO_3_ 0.03 g/L, CoCl_2_·6H_2_O 0.05 g/L, Cu-EDTA 0.4 g/L, CuCl_2_·2H_2_O 0.6 g/L, NiCl_2_·6H_2_O 0.002 g/L, and Na_2_MoO_4_·2H_2_O 0.05 g/L), phosphate buffer (KH_2_PO_4_ 54.4 g/L, Na_2_HPO_4_·12H_2_O 143.4 g/L) 2 mL/L, 4.5 mL/L of a 1 M solution of NaHCO_3,_ and 0.5 mL/L of 1 M Na_2_CO_3_; these were sterilized by filtration and added before use [40]. Liquid cultures were grown in a 500 mL baffled flask sealed with a screw cap in an atmosphere of 30%(v/v) methane in air or 0.2%(v/v) of methanol at 30 °C. Kanamycin was used at a final concentration of 50 μg/mL.

**Table 1:** Strains and plasmids used in this study

Strain or plasmid	Characteristics	Reference or source
Strains		
*E. coli* DH5α	F– endA1 glnV44 thi-1 recA1 relA1 gyrA96 deoR nupG purB20 ϕ80dlacZΔM15 Δ(lacZYAargF) U169, hsdR17(rK–mK +), λ–	Solgent
*M. alcaliphilum* 20Z	Wild-type strain 20Z	DMSZ
20ZE	Evolved putrescine-tolerant strain *M. alcaliphilum* 20Z	This study
20ZES1	Evolved putrescine-tolerant strain ΔspeE1	This study
20ZE3	Evolved putrescine-tolerant strain ΔldhΔackΔspeE1	This study
20ZE4A	Evolved putrescine-tolerant strain ΔldhΔackΔspeE1::argDJ	This study
Plasmid		
pAWP89	pAWP78 containing dTomato driven by pTac promoter	[49]
pCM433KanT	A broad host-range sacB-based vector for unmarked allelic exchange, Kmr	[13]
pCSE1	pCM433kanT containing the deletion construct of speE1	This study
pCM433ldh	pCM433kanT containing the deletion construct of ldh	[16]
pCM433ack	pCM433kanT containing the deletion construct of ack	[16]
pCM351	pCM184 with aaaC1 from pCM350; allelic exchange vector	[50]
pCAR2	pCM351 containing two flanking regions to replace the speE2 by pTac-argDJ fragment	This study
pACE	pAWP89 carrying speC (*E. coli*) and potE driven by tac promoter	This study
pACO	pAWP89 carrying speC (*M. trichosporium* OB3b) driven by tac promoter	This study

### Genetic tools and plasmid construction

All the plasmids in this study were constructed using a Gibson Assembly (NEB, England). Primers were designed using Primer 3 software and synthesized by Macrogen (Seoul, South Korea) as listed in Table 2. Genomic DNA of *E. coli* K12 strain W3110 and *M. alcaliphilum* 20Z were isolated using a Wizard^®^ genomic DNA purification kit (Promega, USA). PCR was performed using Lamp Pfu polymerase (BioFACT, Korea). Plasmids were constructed using genome compiler software. A codon usage table was calculated from nucleotide coding sequences using Emboss cusp [41]. The codon adaptation index (CAI) [42] was calculated using a CAI calculator [43]. All the plasmids and primers used in this study can be found in Tables 1 and 2, respectively. Vector pAWP89 and vector pCM433KanT were linearized by PCR, and vector pCM351 was linearized by a restriction enzyme. Ornithine decarboxylase constitutive (speCEc), putrescine transporter (potE), *N*-acetylornithine transaminase (argD) and ornithine acetyltransferase (argJ) from *E. coli* K12 strain W3110, and ornithine decarboxylase (speCOb) from *M. trichosporium* OB3b were amplified and assembled with the corresponding linearized backbone. The sacB counter-selection plasmids pCSE1 were constructed to delete spermidine synthase (speE1) through unmarked allelic exchange by amplifying and assembling two flanking homology regions upstream and downstream of the target gene. The pCAR2 vector was constructed by cloning the flanking region F2 in the *Sac*I site, and the flanking region F1 and the fragment pTac-argDJ in the *Eco*RI and *Not*I sites.

**Table 2:** Primers used in this study

Primers	Primer sequence 5′ → 3′
pAWP89-ptac-For	TAGTTGTCGGGAAGATGCGT
pAWP89-ptac-Rev	AGCTGTTTCCTGTGTGAATA
FWD_speCEc	cacacaggaaacagctATGAAATCAATGAATATTGCC
REV_speCEc	gtgaatacctTTACTTCAACACATAACCGT
FWD_potE	tgttgaagtaa**AGGTATTCACACAGGAAACAGCT**ATGAGTCAGGCTAAATCGA
REV_potE	gcatcttcccgacaactaTTAACCGTGTTTATTTTTCAGT
FWD_speCOb	ttcacacaggaaacagctATGACCGATCGTATCCGCGAA
REV_speCOb	gcatcttcccgacaacta TCAGATCACGAAGGATTCGAG
FWD_speE F1	tggtctgacagttaccaGCCGGAAATGATGAAGTCCA
REV_speE F1	gggatttctTGCGCATTAGTTATCGAGGAG
FWD_speE F2	taatgcgcaAGAAATCCCTGCAGTGTTGA
REV_speE F2	ccgacaacctgcacatTTCGTTTAAGGTCTGTGCGA
FWD_433speEc	TGTTGCCTTGTGCCGACAT
REV_433speEc	ACCAACACCGGTAACGATAG
FWD_argD	aggtattcacacaggaaacagctATGACCGGCCACATTATGC
REV_argD	tgaatacctCTAGGGTTCATTGGCTTGATG
FWD_argJ	tgaaccctag**AGGTATTCACACAGGAAACAGCT**ATGGCGGTGGGGCAGGT
REV_argJ	gcatcttcccgacaactaCTAAGTCCGATACTCCGCAT
FWD_pTac-argDJ	atgtgcttcTCACATGTTCTTTCCTGCGT
REV_pTac-argDJ	atgctatacgaagttatgcTCGAACTTTTGCTGAGTGGA
FWD_351argDJ_F1	acctgacgtctagatctgCGGCTACAACGTTCTGAAAG
REV_351argDJ_F1	gaagcacatGAAGCACATTCGAGCCTGAA
FWD_351argDJ_F2	gtgttaaccggtgagctTGTTTCAGGATGTCGCTTGG
REV_351argDJ_F2	ggatcctctagtgagctATGTTTAAAAGCTTCGCGGC
FWD_351c	CATGACTTCATGAACGATGA
REV_351c	TCCGACCAATAATCGTAATG

Homologous sequences for Gibson assembly are in lowercase; ribosome binding sites are in bold

### Transformation of *M. alcaliphilum* 20Z strains

The *M. alcaliphilum* 20Z competent cell was prepared as follows. First, 50 mL of *M. alcaliphilum* 20Z strain was grown to an OD_600_ of 0.4–0.6 in a 500 mL flask with 0.2%(v/v) methanol. Then, the cells were harvested and washed twice with cold sterile water by centrifugation at 5000*g* and 4 °C for 10 min. The resulting cell pellets were resuspended in cold sterile water, and 50 µL of the cell suspension were used for electroporation. The competent cells were gently mixed with 500 ng DNA plasmids, and the mixture was then transferred to an ice-cold 1 mm cuvette (Bio-Rad). Electroporation was performed using a Gene Pulser Xcell™ Electroporation system (Bio-Rad) set at 1.3 kV, 25 μF and 200 Ω [13]. After electrical discharge, 1 mL of room temperature NMS medium was added to resuspend the mixture, and then transferred into 20 mL medium in 250 mL serum bottles with 0.2% (v/v) methanol. The cells were incubated at 30 °C for 24 h, and then the cells were harvested and spread onto selective plates in an atmosphere of 30% (v/v) methane in air for 2 weeks.

### Genome-scale modeling

Genome-scale metabolic models of *M. alcaliphilum* 20Z [14] were used to perform a flux balance analysis using Optflux 3 [44]. Necessary reactions of putrescine biosynthesis, and exchange reactions were added to the model, resulting in the i20ZRP model. Engineering strategies were generated by performing Optgene, i.e., the “evolutionary optimization” algorithm, on the i20ZRP model [16].

### Analytical methods

Cell growth was monitored by measuring the optical density at 600 nm with a Beckman spectrophotometer using 1.5 mL cuvettes with a 1 cm path length. Methane concentration was analyzed via gas chromatography (GC; Young In Chromass Corporation, Korea) [45]. Putrescine concentration was analyzed using high-performance liquid chromatography (HPLC; Jasco Corporation, Japan) equipped with a Symmetry C18 column (5 μm, Waters corporation, Massachusetts, USA) operated at 30 °C. A mobile phase (0.7 mL/min) was used for all separations with a UV spectrophotometric detector at 198 nm. Putrescine was first derivatized using a benzoyl chloride pre-column derivatization method [46]. To 500 μL of sample supernatant, 150 μL of 2 N NaOH was added to adjust the pH, followed by 5 μL of benzoyl chloride. The mixture was magnetically stirred for 20 min to complete the reaction. After mixing, 200 μL of saturated NaCl was added. The resulting mixture was extracted by 300 μL ethyl ether. Pooled ether fractions were evaporated by a stream of nitrogen, and the residue was dissolved in 200 μL 42% acetonitrile/water. Then, 40 μL of the resulting solution was subjected to HPLC separation. The standard putrescine was prepared in water and treated as described above.

### Transcriptome analysis

The engineered strain 20ZE4A-pACO was grown in NMS up to OD_600_ of 0.9, and then cells were transferred to NMS and ANMS medium. After 12 h of incubation, cells were harvested for RNA extraction using Qiagen RNeasy kit. Transcriptome shotgun sequencing (RNA-seq) of the engineered strain in these media was performed using Illumina platform (Macrogen Co., Korea). Sequencing reads were aligned with a reference genome and assembled into full-length transcripts, and then gene expression level and different transcript-level expression of different experimental conditions were calculated [47, 48].

## Additional file

**Additional file 1: Table S1.** Knockout strategies suggested by OptGene algorithm for the production of putrescine. **Figure S1.** Knockout of speE1 check by PCR using primer outside of the flanking region. Lane 1 is the mutant strain and Lane 2 is the wild-type strain. **Figure S2.** The insertion of pTAC-argDJ into the genome of the engineered strain 20ZE4A. Lane 1 is the 1 kbp marker Enzynomics, Lane 2 is the wild-type strain, and Lane 3 is the engineered strain.

## Data Availability

The datasets used and/or analyzed during the current study are available from the corresponding author on reasonable request.
