# Supplementary material for: Biological conversion of methane to putrescine using genome-scale model-guided metabolic engineering of a methanotrophic bacterium Methylomicrobium alcaliphilum 20Z
Source: Biotechnol Biofuels. 2019 Jun 15;12:147. doi: 10.1186/s13068-019-1490-z (PMC6570963; doi:10.1186/s13068-019-1490-z)
Supplement: Supplementary file 1 — Additional file 1: Table S1. Knockout strategies suggested by OptGene algorithm for the production of putrescine. Figure S1. Knockout of speE1 check by PCR using primer outside of the flanking region. Lane 1 is the mutant strain and Lane 2 is the wild-type strain. Figure S2. The insertion of pTAC-argDJ into the genome of the engineered strain 20ZE4A. Lane 1 is the 1 kbp marker Enzynomics, Lane 2 is the wild-type strain, and Lane 3 is the engineered strain. [file 13068_2019_1490_MOESM1_ESM.docx]

**Table S1. Knockout strategies suggested by OptGene algorithm for the production of putrescine**

| **Solution** | **BPCY** | **Knockout reactions** |  | **Maximum specific growth rate (h^-1^)** | **Putrescine productivity (mmol/gDCW/h)** |
| --- | --- | --- | --- | --- | --- |
| 1 | 0.0024 | GlyA, ACKr |  | 0.0325 | 0.6572 |
| 2 | 0.00239 | ACKr, MTHFC |  | 0.0324 | 0.6579 |
| 3 | 0.00239 | ACKr, MTHFD |  | 0.0324 | 0.6579 |
| 4 | 0.00238 | ACKr, FTHFLi |  | 0.0320 | 0.6603 |
| 5 | 0.00237 | ACKr |  | 0.0320 | 0.6607 |
| 6 | 0.00199 | NTRIRx, ACKr, CMPA, TPI |  | 0.0461 | 0.3848 |
| 7 | 0.00199 | NTRIRx, ACKr, TPI |  | 0.0470 | 0.3762 |
| 8 | 0.00171 | MTHFD, NADTRHD, AKGDH |  | 0.0697 | 0.2186 |
| 9 | 0.00171 | SUCOAS, NADTRHD |  | 0.0707 | 0.2151 |
| 10 | 0.00171 | NADTRHD, AKGDH |  | 0.0709 | 0.2147 |
| 11 | 0.00171 | GLYA, PTAr |  | 0.0710 | 0.2142 |
| 12 | 0.0017 | GLYA, X5PPKT |  | 0.0731 | 0.2070 |
| 13 | 0.0017 | PTAr, FTHFLi |  | 0.0733 | 0.2065 |
| 14 | 0.0017 | MTHFD, X5PPKT |  | 0.0733 | 0.2064 |
| 15 | 0.0017 | MTHFC, X5PPKT |  | 0.0733 | 0.2064 |
| 16 | 0.0017 | NTRIRy, PTAr |  | 0.0736 | 0.2054 |
| 17 | 0.0017 | X5PPKT |  | 0.0740 | 0.2042 |
| 18 | 0.0017 | GLYA |  | 0.0741 | 0.2036 |
| 19 | 0.00169 | MTHFC |  | 0.0743 | 0.2030 |
| 20 | 0.00169 | MTHFD |  | 0.0743 | 0.2030 |


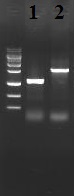


**Figure S1.** Knockout of speE1 check by PCR using primer outside of the flanking region. Lane 1 is the mutant strain and Lane 2 is the wild-type strain.


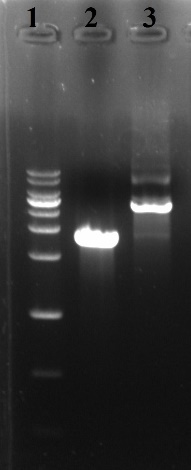


**Figure S2.** The insertion of pTAC-argDJ into the genome of the engineered strain 20ZE4A. Lane 1 is the 1 kbp marker Enzynomics, Lane 2 is the wild-type strain, and Lane 3 is the engineered strain.
